# Bilateral acute angle closure after venlafaxine intake in a young patient with a narrow iridocorneal angle

**DOI:** 10.22336/rjo.2021.56

**Published:** 2021

**Authors:** Ana Isabel Ramos Castrillo, María Larrañaga Cores, Mercedes Giménez de Azcárate Reyes, Eva Fernández Gutiérrez, Claudia Klein Burgos

**Affiliations:** * Glaucoma Unit, Ophthalmology Service, La Paz University Hospital, Madrid, Spain; ** Ophthalmology Service, La Paz University Hospital, Madrid, Spain

**Keywords:** angle closure, acute glaucoma, venlafaxine, selective serotonin, noradrenaline reuptake inhibitors

## Abstract

We report a case of bilateral and acute angle closure after a single dose of antidepressant venlafaxine in a 40-year-old woman with no previous pathologies, who asked for consultation for blurred vision and pain in the left eye. Initial evaluation included visual acuity, slit lamp biomicroscopy and intraocular pressure (IOP) measurement using Goldmann’s applanation tonometer. Gonioscopy and fundus examination were also performed in both eyes. Examination and IOP supported the diagnosis of acute glaucoma in the left eye. The patient’s evolution was satisfactory after bilateral peripheral iridotomy was performed with Nd-YAG laser, as described in the cases reported in the international literature. The pathophysiology of angle closure and its relationship with venlafaxine intake were also discussed.

## Introduction

Antidepressants are drugs frequently prescribed in the normal clinical practice. Among them, tricyclics are used less and less due to their side effects, and have been replaced by new pharmacological families with a better safety profile, such as selective serotonin reuptake inhibitors or serotonin and noradrenaline reuptake inhibitors [**1**].

However, the increasingly widespread use of these new antidepressants leads to a higher incidence of also important side effects, such as acute glaucoma. A review of literature reveals case reports linking these antidepressant families and angle closure. However, the symptoms are sometimes subtle and go unnoticed. Furthermore, the distribution of the different iridocorneal angle configurations in the general population implies that the adverse effect leading to angle closure could be poorly symptomatic in most patients (intermittent, subacute, or progressive angle-closure glaucoma). As a result, the incidence is likely to be underestimated [**2**]. 

Venlafaxine is a serotonin and norepinephrine reuptake inhibitor (SNRI). Although listed as an adverse reaction, glaucomatogenic potential has rarely been documented with a therapeutic dose of venlafaxine. We present the case of a 42-year-old woman who presented with bilateral acute angle closure after taking a single therapeutic dose of venlafaxine.

## Clinical case

We present the case of a 42-year-old woman with no history of allergic drug reactions or previous illnesses of interest, who came to the emergency room with blurred vision in both eyes and pain in the left eye since the afternoon of the previous day. She also presented nausea and general malaise. She admitted having taken 1 venlafaxine tablet 2 hours before the onset of symptoms. She did not admit having taken any other drug.

During the anamnesis, the patient reported episodes of blurred vision, headache, and a sensation of eye pressure at the end of the working day and in scotopic vision conditions on previous occasions.

In the ophthalmological examination in the emergency room, visual acuity without correction at the time of the visit was ½ (improvement with use of pinhole occluder to 2/ 3) in the right eye; and visual acuity with the left eye was counting fingers at 50 cm (without improvement with the use of pinole occluder). Slit lamp biomicroscopy showed a 2+ conjunctival-ciliary mixed injection, with corneal stromal edema in the left eye. The anterior chamber was constricted in both eyes. Tyndall was not appreciated. The pupil was in poorly reactive middle mydriasis in the right eye and arreactive in the left eye. The patient had incipient phacosclerosis in both eyes. IOP was measured with the Goldmann applanation tonometer, being 10 mmHg in the right eye and 32 in the left eye. Gonioscopy was performed with and without decentration, which revealed a degree 0 angle closure in the 4 quadrants in both eyes, without observing peripheral anterior synechiae. The fundus showed normoexcavated, normocoloured papillae with defined edges and structured macules without vascular or parenchymal alterations.

The diagnosis was bilateral primary angle closure with concomitant acute glaucoma in the left eye, secondary to previous venlafaxine intake.

The standard medical treatment regimen for acute angle closure glaucoma was started immediately and peripheral iridotomy with Nd-YAG laser was performed in both eyes. At 30 minutes after iridotomy, the patient had less corneal edema and the IOP measured with the Goldmann applanation tonometer was 10 mmHg in both eyes. After one-week, visual acuity was 1 in both eyes, and IOP remained within normal limits. Venlafaxine treatment was suspended.

The patient consented to publication of the case in writing. 

## Discussion

Many commonly prescribed drugs have optic nerve damage as a potential secondary effect, glaucoma (both primary and secondary) being one of its forms. Most of the drugs with potential glaucoma risk are related to the induction of angle closure [**3**], more easily in patients with predisposing anatomical factors (narrow anterior chamber, plateau iris configuration, short axial length, etc.). Some of the drugs that would be included in this list of risk of angle closure development are: adrenergic agonists, anticholinergics, sulfonamides, or serotonergic agents, being the first two groups that cause pupillary blocks (it is this pupillary block that leads to angle closure, and thus the development of acute glaucoma is favored) most frequently [**4**]. 

There are other mechanisms by which drugs can cause damage to the optic nerve in the form of glaucoma. Corticosteroids and some antineoplastic agents have been linked to the development of open-angle glaucoma, and sulfonamides have done so with an idiosyncratic reaction [**3**]. 

Regardless of the mechanism, the first thing a doctor should do when suspecting glaucomatous damage because of taking a drug is to discontinue treatment with that drug.

Venlafaxine, an antidepressant drug, belongs to the group of serotonin and norepinephrine reuptake inhibitors, its action on serotonin reuptake being more powerful. Although glaucoma is a rare complication of venlafaxine treatment [**5**], more and more cases are being reported, which makes us reconsider its safety profile and forces us to further study the still unknown production mechanism. Some of the hypotheses proposed for this mechanism are mydriasis that leads to a thickening of the base of the iris or supraciliary effusion that produces an anterior rotation of the ciliary body [**4**,**6**-**9**]. In both cases, this would lead to a potential angle closure, but cases of open angle glaucoma associated with venlafaxine intake have also been documented [**9**].

In the case of other SSRI drugs related to glaucomatous damage, such as paroxetine, the cause was postulated to be either the anticholinergic or serotonergic effects produced by this group of drugs [**6**]. Since venlafaxine has no anticholinergic effects, this could support the hypothesis that the damage is most likely due to the effects produced by accumulation of serotonin. Another alternative would be the weak adrenergic effects associated with this drug [**6**]. 

Some drugs with serotonergic activity are: serotonin reuptake inhibitors (SSRIs), serotonin and norepinephrine reuptake inhibitors (SNRIs), monoamine oxidase inhibitors (MAOIs), tricyclic antidepressants, triptans, etc. Stimulation of receptors of serotonin type 5-HT7 produces a relaxation of the iris sphincter, mydriasis, and an increase in the production of aqueous humor. These receptors are present in the ciliary body, trabecular meshwork, choroid, and sphincter of the iris. Furthermore, SNRIs also have adrenergic and dopaminergic activity, thereby also producing mydriasis and increased production of aqueous humor [**4**,**9**]. 

The increase in glaucoma cases reported in relation to the taking of SNRI drugs such as venlafaxine makes us consider its production mechanism, with the aim of making both a diagnosis and an early treatment [**1**,**6**-**8**,**10**]. In cases in which acute angle closure occurs, the first measure to take would be immediate drug withdrawal and peripheral iridotomy. However, we must consider, as we say, the possible production mechanisms and that not all cases will be resolved with this measure (for example, when the cause is uveal effusion) [**4**].

## Conclusion

Primary angle closure is a possible and potentially serious complication of the administration of certain drugs. The prior knowledge of this possible complication and the study of the mechanisms by which it occurs can help the doctor to make both a diagnosis and an early treatment, thus improving the prognosis.

By presenting this clinical case, we proposed the benefit of also informing the patient about the alarm symptoms for which they should promptly consult an ophthalmologist when taking SNRI or SSRI.

**Conflict of Interest statement**

The authors declare that they have no conflict of interest.

**Informed Consent and Human and Animal Rights statement**

The patient consented to the publication of the case. 

**Authorization for the use of human subjects**

Ethical approval: The research related to human use complies with all the relevant national regulations, institutional policies, is in accordance with the tenets of the Helsinki Declaration, and has been approved by the review board of La Paz University Hospital, Madrid, Spain.

**Acknowledgements**

None.

**Sources of Funding**

No funding or grant support.

**Disclosures**

None.
